# Supramolecular Self-Assembly of a Model Hydrogelator: Characterization of Fiber Formation and Morphology

**DOI:** 10.3390/gels2040027

**Published:** 2016-10-08

**Authors:** Yuan Gao, Ryan Nieuwendaal, Emilios K. Dimitriadis, Boualem Hammouda, Jack F. Douglas, Bing Xu, Ferenc Horkay

**Affiliations:** 1 Section on Quantitative Imaging and Tissue Sciences, Eunice Kennedy Shriver National Institute of Child Health and Human Development, National Institutes of Health, Bethesda, MD 20892, USA; 2 Materials Science and Engineering Division, National Institute of Standards and Technology, Gaithersburg, MD 20899, USA; ryan.nieuwendaal@nist.gov; 3 NIST Center for Neutron Research, National Institute of Standards and Technology, Gaithersburg, MD 20899, USA; hammouda@nist.gov; 4 National Institute of Biomedical Imaging and Bioengineering, National Institutes of Health, Bethesda, MD 20892, USA; dimitre@helix.nih.gov; 5 Department of Chemistry, Brandeis University, Waltham, MA 02453, USA; bxu@brandeis.edu

**Keywords:** hydrogel, fiber formation, small angle neutron scattering, dynamic light scattering, NMR, rheology

## Abstract

Hydrogels are of intense recent interest in connection with biomedical applications ranging from 3-D cell cultures and stem cell differentiation to regenerative medicine, controlled drug delivery, and tissue engineering. This prototypical form of soft matter has many emerging material science applications outside the medical field. The physical processes underlying this type of solidification are incompletely understood, and this limits design efforts aimed at optimizing these materials for applications. We address this general problem by applying multiple techniques (e.g., NMR, dynamic light scattering, small angle neutron scattering, rheological measurements) to the case of a peptide derivative hydrogelator (molecule **1**, NapFFKYp) over a broad range of concentration and temperature to characterize both the formation of individual nanofibers and the fiber network. We believe that a better understanding of the hierarchical self-assembly process and control over the final morphology of this kind of material should have broad significance for biological and medicinal applications utilizing hydrogels.

## 1. Introduction

There has been much recent work in synthesizing materials through ‘bonds’ created by molecular association rather than permanent chemical bonds. Such ”supramolecular” materials [1,2] are the norm in biological systems and this type of material is becoming increasingly important in numerous fields ranging from diverse agricultural products, food additives to drug delivery systems, and separation of biomacromolecules [3,4,5,6]. In the present work, we focus on hydrogels formed by molecular association of small ”gelator” molecules that self-assemble into fiber networks with gel-like properties [7]. Although supramolecular hydrogel formation has many proven applications [8,9], including cell culture [10], tissue engineering [11,12,13], and regenerative medicine [14,15], there is limited understanding of the factors that control the nanoscale structure and stability of these materials. A better understanding of the effect of structure and interactions of the individual building blocks on the properties of the resulting assemblies is required for the design of supramolecular biomaterials tailored to specific applications in biology and medicine. There have been many previous studies of peptide-based hydrogels and Yan and Pochan [16] and Chow et al. [17] have recently reviewed this field.

Although there have been many previous studies of the formation of thermally reversible gels from small molecule “gelators” [18,19] both in aqueous and organic solvents, many issues regarding the underlying physical interactions and molecular processes by which the gels form remain unclear. The early stage of gel formation is particularly hard to study because of the limited number of experimental methods having sufficient time and spatial resolution. Scattering techniques, such as small-angle neutron and light scattering in both static and dynamic modes, have the required resolution and offer a potential window into the early stages of gel formation and the fine structure of gel morphology, which complement microscopy and rheology measurements on thermally reversible gels. In the present work, we study gel formation using a model hydrogelator through a combined scattering, imaging, spectroscopic (nuclear magnetic resonance) and rheological characterization to obtain a multi-scale view of gel formation and morphology. This particular gelator molecule has been the focus of several previous studies [20,21,22,23,24] because of its unusual property of passing through the cell membranes of HeLa cells and gelling in the cytoplasm when coming in contact with certain enzymes, an effect of potential medical significance. However, in the present work, we focus on the association of this molecule in solution in the *absence of enzyme* where the binding is thermally reversible. In this context, this model hydrogelator is suitable for studying essential physical aspects of aqueous thermoreversible gelation and, indeed, we find that the gelation process and gel morphology appear to be quite similar to peptide and protein gelators of great importance in medical and manufacturing applications.

The paper is organized as follows. First, the morphology of hydrogels and nanofibers determined by AFM and TEM are presented. This is followed by the construction of a diagram that delineates the qualitative rheological states of our system (simple fluid, viscoelastic fluid, gel) as a function of the gelator concentration and temperature. We then refine our qualitative characterization based on the gross fluid flow properties by NMR and rheological measurements that quantify the extent of gelator molecule association at equilibrium and rheological state. This information allows us to design our scattering and imaging measurements to address different stages of gel assembly. In particular, we first probe the pre-gel viscoelastic gel by small angle neutron scattering (SANS) and dynamic light scattering (DLS) into the relatively poorly understood incipient stage of gel formation. We then apply these scattering methods, along with direct imaging by AFM and TEM in the fully developed gel regime to achieve a multi-scale description of both the structure and dynamics of these complex materials. Finally, the sample preparation and the measurement techniques (nuclear magnetic resonance (NMR), dynamic light scattering (DLS), small angle neutron scattering (SANS), transmission electron microscopy (TEM), atomic force microscopy (AFM), rheology) are described.

## 2. Results and Discussion

### 2.1. Morphology of Nanofibers

**Scheme 1:**
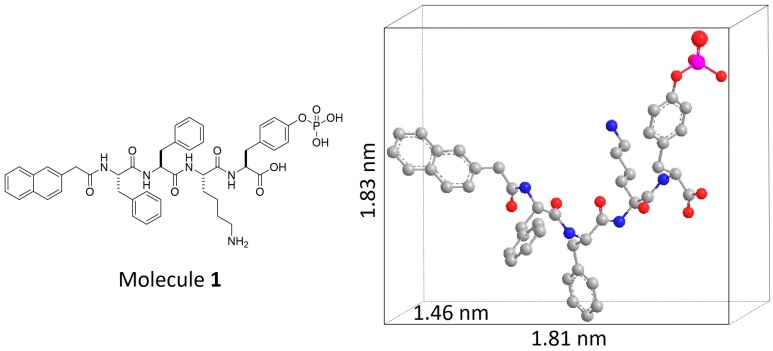
Structure of molecule **1** (chemical name: (2*S*,5*S*,8*S*,11*S*)-5-(4-aminobutyl)-8,11-dibenzyl-14-(naphthalen-2-yl)-4,7,10,13-tetraoxo-2-(4-(phosphonooxy)benzyl)-3,6,9,12-tetraazatetradecanoic acid) and its physical dimension. Molecular dimensions are indicated by a box enclosing the molecule. Grey: carbon; Red: oxygen; Blue: nitrogen; Purple: phosphorus while hydrogen is omitted.

**Figure 1:**
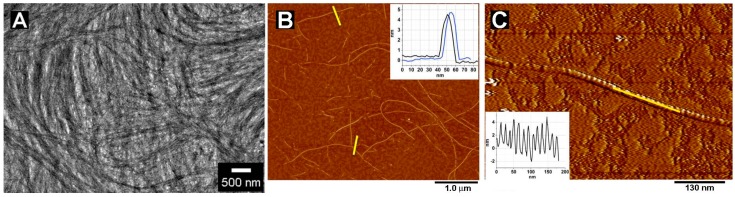
(**A**) Transmission electron microscopy image represents the typical micro-morphology of 5.0 mass % of hydrogel **1**; (**B**) Atomic force microscopy image of nanofibers in the hydrogel **1** at the concentration of 5.0 mass % (mica was pre-coated with 5 mM of MgCl_2_ solution). Due to the different sample preparation procedures (sample rinsing, etc.) the number density of nanofibers is greatly altered, but the basic fiber morphology is largely preserved. Inset shows sections of the fibers at two locations (scan positions indicated by yellow lines); (**C**) Zoomed-in image of a fiber length (phase channel) and inset showing a section along the fibers axis (shown by yellow line) clearly exhibiting a periodic pattern that we interpret to arise from fiber twisting.

Figure 1 shows the morphology of a typical hydrogel formed from nanofibers composed of molecule **1** (see Scheme 1).

In the gel, the nanofibers are very long, polydisperse, and cannot cross each other, so that the viscoelastic relaxation time is effectively infinite [25]. Thus, the whole sample is able to sustain its initial shape and to resist flowing when subjected to weak shearing forces. The TEM image (Figure 1A) represents the morphology of a dense nanofiber network. TEM imaging shows a projection of the objects and thus provides little information about the internal structure of nanofibers [26]. Nonetheless, TEM gives a good qualitative idea of the structure of the self-assembled fibers in their fully mature form.

AFM is a real space imaging method for investigating the morphology of nanofibers. Figure 1B displays the AFM image of nanofibers similar to those shown in Figure 1A, but the fibers are in a more dilute state due to the method of sample preparation, which involved rinsing the sample to remove excess material (as described below in the experimental section). The fibers appear to be flattened on the substrate as the ratio of width/height is ≈3.5. This is probably caused by a combination of adhesion forces and of the force applied by the probe during scanning. However, assuming fiber incompressibility, we can estimate the cross-sectional area of the fibers and from that, compute an equivalent radius for a circular fiber. The insert in Figure 1B shows two cross-sections of such fibers. The width is measured at half-height and the cross-section area is computed as that of an ellipsoid with half-height and half-width being the two radii of the ellipse. The equivalent circular radius is then, *r* = ½ √*h* *w*, where *h* and *w* are the height and width of the fiber in the AFM image, respectively. Based on this model of the fibers, we estimate the fiber radius to equal, *r* = 3.6 ± 0.2 nm. This finding suggests that the thickness of the fibers is about 4–6 times greater than the size of the individual gelator molecules. In addition, the fibers evidently exhibit a periodic pattern along their axis with periodicity of ≈14.5 nm, as is clearly seen in the phase image of a longitudinal section of a fiber in Figure 1C. This patterning is interpreted to arise for a periodic twisting of the fiber. Below, we explore the fiber thickness and inter-fiber spacing using neutron scattering, a method that does not perturb the gel morphology due to sample preparation, such as sample washing and film evaporation before the AFM and TEM measurements are performed.

### 2.2. Characterization of the Rheological Behavior

The stability and geometrical properties of the fiber network depend on the temperature and gelator concentration and can also be dependent on sample history. First, we establish the ‘rheology diagram’ of our hydrogelator, which will be a basic reference point for the neutron scattering and NMR measurements described below. This is the natural first step in the characterization of any hydrogel. Within each regime, we employ further techniques, such as microscopy, dynamic light scattering, small angle neutron scattering to extract structural information (fiber diameter and shape, persistence length, mesh size/correlation length, fiber length), and relaxation time as a function of temperature and gelator concentration. We consider a rough rheological estimate of solution conditions, where the solution is a simple fluid, a viscoelastic fluid, and then a solid characterized by a complete lack of apparent flow over an appreciable time scale. Glass vials containing gelator solutions were incubated in a water bath at different temperatures. Each sample was allowed to equilibrate for at least 30 min before we tilted the glass vials at a 45-angle to determine if the sample could maintain its shape against gravity (a poor man’s test of gelation). The simple “tilt the vial” test yields qualitative information on the sol-gel transition that helps in the design of more precise measurements characterizing the gelator assembly and gel.

**Figure 2:**
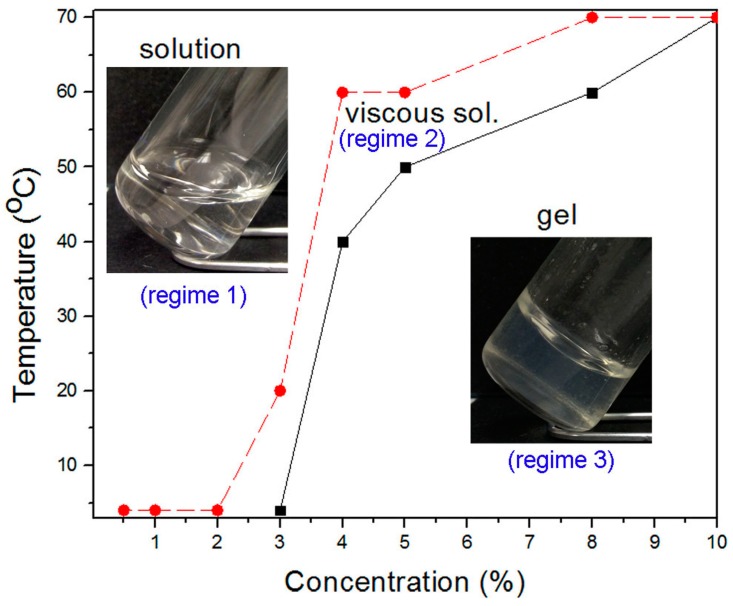
Phase diagram of **1** in water at pH = 7 showing the three regimes: solution, viscous solution, and gel.

As shown in Figure 2, the gelator is in the ‘sol’ or fluid state at mass concentrations below 2 mass % over the whole temperature range explored here. Measurements in the pre-gel regime (simple fluid and viscoelastic fluid regimes) provide information about the geometry of the proto-filament assemblies, in other words, the precursor morphology to the fibers shown in Figure 1. At concentrations between 3 mass % and 10 mass %, the gel transforms into a liquid upon heating. It is likely that the gelator molecules exhibit significant association in the solution in this pre-gel regime, as evidenced by the relatively high viscosity and hazy appearance of the solution. The 5 mass % sample became very viscous below 50 °C, and eventually converts to a stable “gel”, defined by the lack of flow over appreciable time scale when the sample vial is tilted. Of course, gels can also be formed by increasing the gelator concentration at constant temperature [8,27]. This phenomenology leads us to define rheological regimes, as indicated in Figure 2.

The transition line separating the sol and gel states is normally a monotonically increasing function of the gelator concentration, and we show this locus for our gelator in Figure 2. We designate the “sol” regime as regime 1, the “gel” regime as regime 3, and we also designate a “viscoelastic fluid” regime 2, obtained by either “melting” the gel by heating [28] or cooling the “sol”. In the intermediate regime 2, the sample fails to maintain its shape as a gel at short times, and flows on long time scales so that the material is a viscoelastic ”jelly”.

The physical nature of the precursor assemblies in regime 1 is one of the least understood aspects of gelator assembly and leads to the question of how and why the fibers form starting from the assemblies found in this regime. Fortunately, this regime is well-suited for small-angle neutron scattering (SANS) and light scattering measurements that can illuminate this initial stage of gelator assembly. Before describing these measurements, however, we refine our understanding of the temperature and concentration range in which gelator assembly occurs using ^1^H-NMR, which allows for estimation of the fraction of gelator molecules in the self-assembled state [29], in other words, the basic order parameter for the assembly process.

### 2.3. Characterization of Thermodynamic State

**Figure 3:**
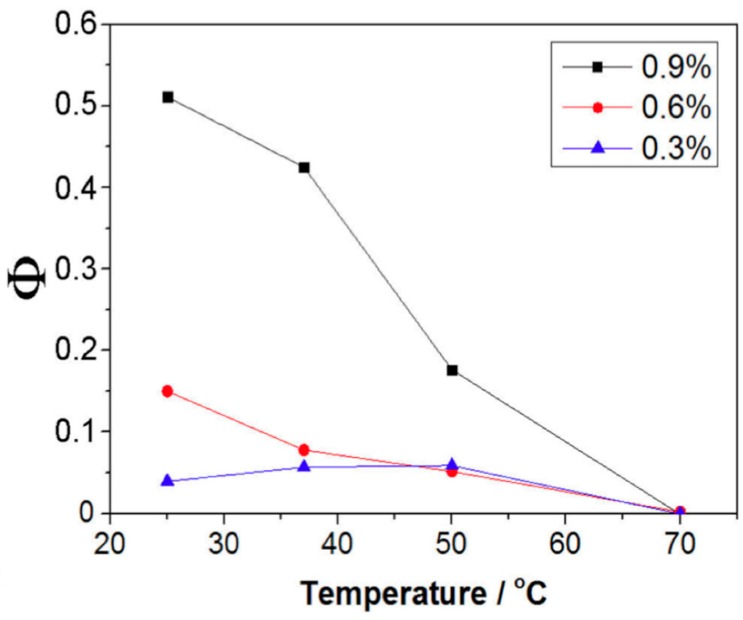
Self-assembled portion of **1** in solutions determined by ^1^H-NMR.

For reversible molecular self-assembly at equilibrium, the extent of assembly Φ (i.e., fraction of gelator in the assembly) [30] depends on the gelator concentration c, temperature, and various physical factors (e.g., salt concentration, pH, etc.) that affect gelator stability. In particular, Φ describes the extent to which the gelator changes from being dispersed in solution to being organized into self-assembled structures and thus provides a basic ‘order parameter’ for the assembly process [31]. This information suggests the temperature (*T*) and concentration (*c*) range that is most suitable for the scattering measurements. Figure 3 shows our estimation of Φ as a function of *T* at three gelator concentrations, based on NMR measurements that we describe below.

The variation of the proton T_2_ time determined from solution NMR measurements as a function of gelator concentration and temperature provides information about the progression of the gelation process. As the gelator (**1**) molecules assemble, they become ‘invisible’ in the NMR spectrum since the spectral window is too narrow (5 to 6 kHz) to detect the broad resonance of the aggregated species (>10 kHz). Hence, after comparing to an internal intensity standard (4,4-dimethyl-4-silapentane-1-sulfonic acid) of known concentration, we calculated the concentration of non-aggregated species. Figure 3 shows the temperature dependent concentration of the ”free” gelator observed by ^1^H-NMR. At a concentration of 0.3 mass %, the concentration of unassociated gelator is almost the same as the total gelator concentration in solution from 25 °C to 70 °C so the gelator shows no evidence for self-assembly in this concentration range. At higher concentrations (0.6 mass % and 0.9 mass %), however, the gelator progressively transforms into assembled state upon cooling. We see that a good temperature range for observing this assembly process is between 30 °C and 50 °C.

Although the extent of gelator association Φ evolves after a temperature jump, it approaches a constant value after the assembled structure comes into equilibrium [32]. However, non-equilibrium effects are sometimes apparent if the self-assembled structure becomes trapped into low potential structures (e.g., highly bundled fibers), making the assembly process essentially non-reversible on any reasonable timescale [33]. Many of the peptide gels are conspicuously non-reversible in their assembly kinetics because of their tendency to not fold in a regular fashion or because of fiber bundling in which there are multiple bonding contacts that must be simultaneously broken in order for the fiber assemblies to disassemble. Pochan and coworkers have introduced design principles to obtain more reversible folding in peptide gelators [34,35,36,37]. Many natural protein gelator molecules of biological significance for cell function have been evolutionarily optimized to exhibit facile reversible fiber formation, such as actin [38,39], while many other assembling systems, such as viral capsids [33], can strongly resist disassembly once formed. The stabilization of self-assembled structures has evident biological ramifications and technological importance in a synthetic assembly context. In the present work, we confine our attention strictly to a physical regime in which the molecular self-assembly process is reversible or nearly so. It just happens that our synthetic gelator exhibits *reversible* self-assembly, an attribute that is highly attractive to model the assembly process. In such systems, it should be possible to *tune* the average size of the assemblies with varying temperature, gelator concentration, salt, or other variable influencing solution thermodynamic conditions.

### 2.4. Rheological Characterization of Gelation Transition

**Figure 4:**
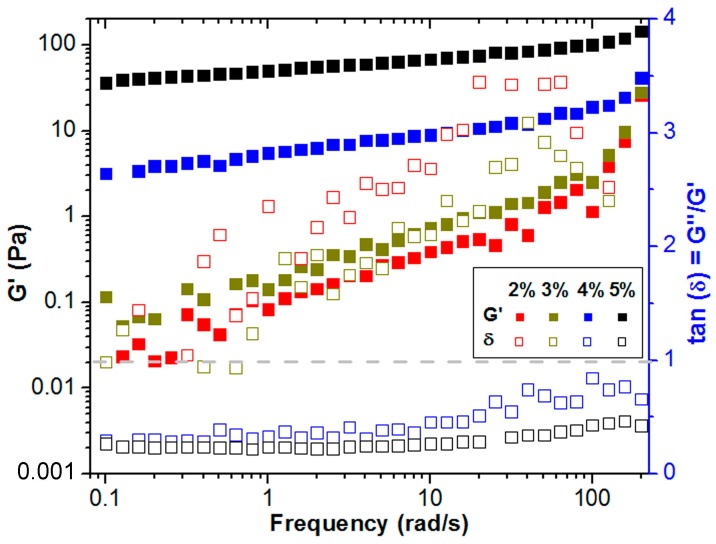
Dependencies of *G’* and tan δ (=*G”*/*G’*) on the frequency at different gelator concentrations. We see a progressive change in the viscoelastic response from a viscoelastic fluid to a solid gel with increasing concentration.

To determine more precisely the transition from the liquid to the ‘gel’ state (see Figure 2 and Figure 3), we made rheological measurements at different gelator concentrations. Rheological measurements allow us to quantify the evolution of the frequency-dependent storage modulus *G’* (Figure 4). At low concentrations (corresponding to regime 1), a liquid like viscoelastic response is observed; *G’* increases with increasing frequency. However, the gelled samples show less frequency dependency than the sol samples. At higher gelator concentrations (above 4 mass %), tan δ = *G”*/*G’* (where *G”* is the loss modulus) becomes smaller than 1 in the frequency window studied, which is characteristic of gels [7]. It is important to note that the moduli of physical gels may exhibit weak frequency dependency due to the transient nature of the cross-links. Of course, this type of thermally reversible sol-gel transition has been observed in numerous other gelator systems [8,9,18,40]. We further probe this emergent elasticity by small angle neutron scattering (SANS) and dynamic light scattering (DLS) measurements below.

### 2.5. Neutron and Light Scattering Characterization of Sol and Gel States

**Figure 5:**
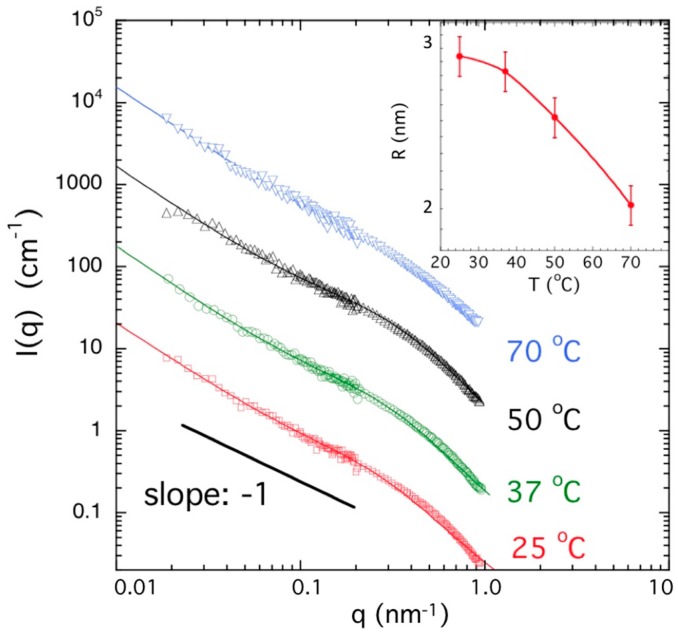
Small angle neutron scattering (SANS) profiles of 0.5 mass % gelator samples at different temperatures. For clarity, the SANS curves are shifted by a factor of 10 with increasing *T*. The continuous lines through the data points are least squares fits of Equation (1). The inset shows the variation of the cross-sectional radius *R* with *T*. Error bars correspond to one standard deviation.

In the sol regime (regime 1), corresponding to low concentration and high *T*, the gelator molecules are well dispersed in the solution. Given the somewhat turbid appearance of the solution in this regime, we may expect structure formation to also arise even in this fluid regime. To obtain information on the geometry of the fiber precursor structures, we performed SANS measurements in regime 1 with the hope of being able to tune the fiber size with *T*. Figure 5 shows the SANS profiles for a 0.5 mass % sample measured at four different temperatures. The SANS data were analyzed based on the relation,

(1) $$I(q)=\frac{B}{\left(1+q^{2}R^{2}\right)}+Cq^{-n}$$

where *R* is the cross sectional radius of the precursor assemblies, *B*, *C*, and *n* are constants. The scattering intensity decreases over the whole *q* range investigated. At low *q*, a slope close to −1 is expected for a solution of scattering stiff fibers. The absence of a plateau region implies that the size of the scattering objects exceeds the resolution of the SANS instrument. However, from the shoulder at high *q*, we can at least estimate the cross sectional radius *R* of the precursor fiber structures. We see from the inset in Figure 5 that *R* gradually decreases with increasing temperature. We interpret this trend based on direct TEM observations by Yucel et al. [41] on early stage fiber growth in another peptide gelator. In particular, they observe that the primary scale of the fiber is directly related to the folded dimension of the peptide molecules, but they also see defects in the fibers that tend to increase the effective fiber diameter.

These fiber defects have critical importance for the gel properties since they give rise to fiber junctions at long times. The approximately 2.0 nm dimension of the fibers that we observe in this regime is consistent with the folded dimension of molecule **1** as shown in Scheme 1 and this value is also comparable to the dimensions of the peptide fibers observed by Yucel et al. Correspondingly, we attribute the larger *R* at lower *T* to progressive branching and defect formation at lower temperatures. The average diameter of the fibers measured by scattering have a size on the order of folded gelator. At temperatures close to the fiber melting temperature, the effective size becomes somewhat larger due to progressive defect formation in the self-assembled fiber network. Progressive defect formation in fiber forming gelator systems is a well- recognized general tendency in this type of gelator and ultimately this type of fiber branching leads to a tendency to form spherulites [42]. In some fibril forming systems, these ”proto-filament” fibers form hierarchical bundles, but in other gelators the fibrils resist this tendency. It is evident from Figure 1 that our peptide gelator does not exhibit a strong tendency towards fiber bundling, which is probably an important factor in its reversibility. We conclude that the morphology of our peptide gelator is similar to peptide gelators studied before by Pochan and coworkers [34].

Having established a rough rheological state diagram and the formation of molecular fibers in regime 1 from SANS, we next consider dynamic light scattering (DLS) measurements on a gelator solution in the boundary between regime 1 and regime 2 to characterize the slowing down of relaxation upon approaching the gel state. The self-assembled structures in this regime should be far too large to probe with neutron scattering. Even for the 0.5% gelator mass sample, the fibers were too large for a quantitative characterization of the overall size precursor fibers. However, light scattering allows us to probe the dynamics at much larger scales than possible with SANS measurements.

**Figure 6:**
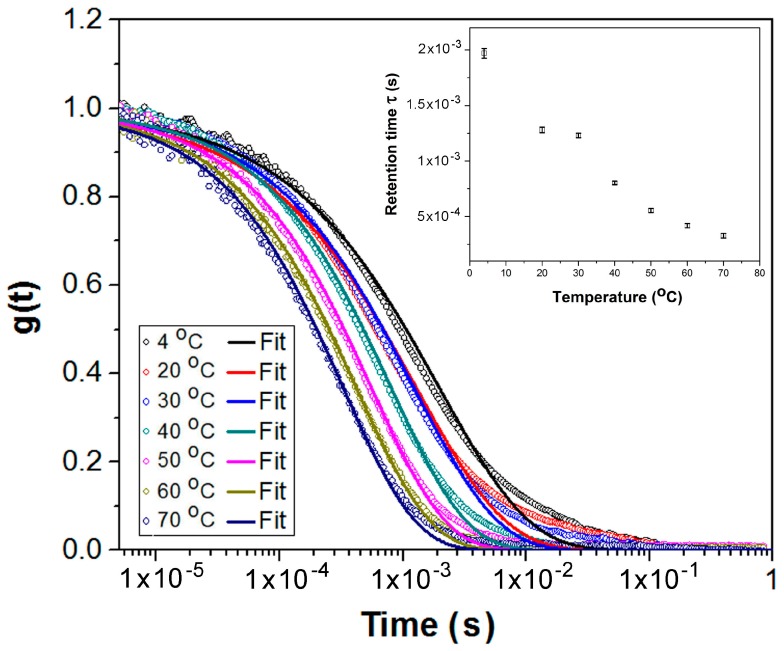
Dynamic light scattering autocorrelation functions g(t) for a 2 mass % gelator sample at different temperatures. The continuous curves through the data points are least squares fits to Equation (2). Inset: Plot of τ vs. temperature. We observe a sharp rise in τ upon cooling.

Figure 6 shows the DLS autocorrelation functions for a 2 mass % gelator sample, corresponding to a regime 1 sample near the border of forming a gel (see Figure 2 and Figure 3). We clearly observe that the DLS signal decays faster at higher temperature, which is consistent with the expected presence of smaller self-assembled structures in the solution. We analyzed the autocorrelation function *g*(*t*) for light intensity fluctuations by a stretched exponential function,

*g*(*t*)/*g*(0) = exp[−(*t*/τ)^α^] (2)

where *t* is the time, τ is the relaxation time, and g(0) and α (≈0.6) are constants. Stretched exponential relaxation is characteristic of polydisperse polymer structures formed by self-assembly [43]. In Figure 6, the continuous lines are the fits of Equation (1) to the experimental data. The inset shows that τ increases with decreasing temperature, which is consistent with a progressive rigidification of the network since the relaxation time is proportional to the gel longitudinal modulus [44].

**Figure 7:**
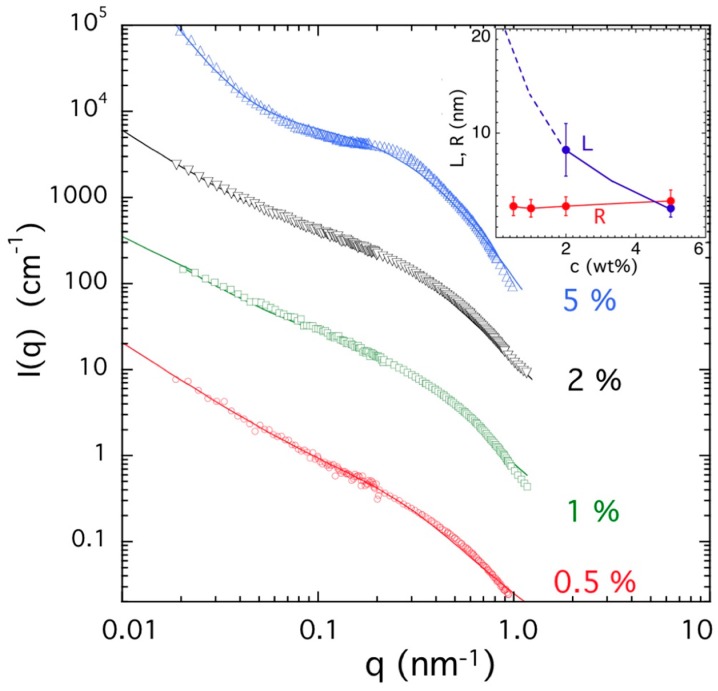
SANS profiles of gelator systems at different concentrations (*T* = 25 °C). For clarity, the curves are shifted by a factor of 10 with increasing gelator concentration. The continuous lines through the data points are least squares fits of Equation (1) (0.5 mass % and 1 mass %) and Equation (3) (2 mass % and 5 mass %). The inset shows the variation of *R* and *L* with the gelator concentration.

We next consider the progressive change in fiber organization as a function of gelator concentration as viewed by SANS, which probes the structure of the hydrogel at a molecular scale. Figure 7 illustrates the SANS profiles at different gelator concentrations spanning the regimes 1 to 3. It is clear from this figure that the curvature of the SANS curves changes with increasing gelator concentration. For high gelator concentrations, we observe a plateau region in the intermediate *q* region. This scattering feature and the linear scaling with *q* in this regime are both often observed in gels having a fibrous fine structure. In particular, this type of scattering is consistent with the formation of a macroscopic branched network of fibers of the form shown in Figure 1a. At low *q*, the slope increase indicates the presence of large scale heterogeneities, a scattering feature that is most pronounced in the 5 mass % sample. In the two low concentration samples, the SANS response can be satisfactorily described by Equation (1), but at higher gelator concentrations (*c* ≥ 2 mass %) in Regime 3, two characteristic length scales are required to fit the data [45],

(3) $$I(q)=\frac{B}{(1+q^{2}R^{2}){\left[1+{\left(qL\right)}^{2}\right]}^{1/2}}+Cq^{-n}$$

where *L* is taken to be the inter-fibrillar spacing between the fibers in the network. We note that in semi-dilute polymer solutions and gels, *L* is termed the ‘mesh size’ of the network. It is often observed in polymer solutions that the mesh-size decreases as the polymer concentration increases. We next examine whether this general trend is exhibited by our gelator.

Our analysis of the SANS curves in terms of Equation (3) shows that *L* significantly decreases with increasing gelator concentration, where this scale emerges at some percolation-like concentration where the network becomes macroscopically spanning. On the other hand, *R*, which we identify with the effective fiber radius, varies rather little (Inset to Figure 7) with gelator concentration. The *R* values in this range are not significantly different from those observed in regime 1 (inset of Figure 5). This finding suggests that the fibrils are less defective in their organization at elevated temperatures approaching the ‘melting’ temperature of the gelator fibers. We note that the value of *R* (=2.9 ± 0.2 nm at 25 °C) from the SANS measurements is in excellent agreement with that estimated from the corresponding AFM imaging (*R* = 3.6 ± 0.2 nm). The difference between the two methods is within noise.

**Figure 8:**
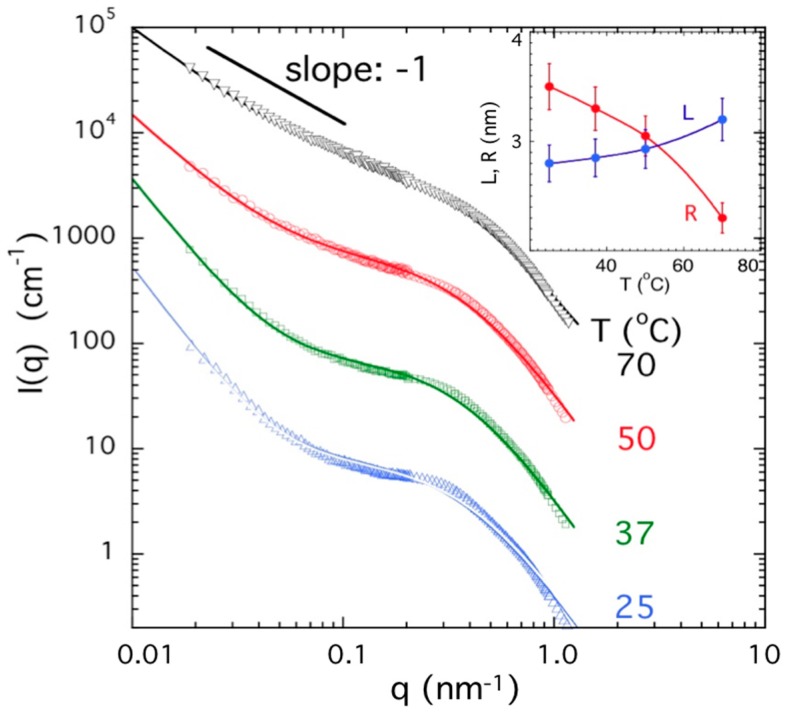
Temperature dependence of the SANS profiles of the 5 mass % gelator sample. For clarity, the curves are shifted by a factor of 10 with increasing temperatures. The continuous lines through the data points are least squares fits of Equation (3). The inset shows the variation of *R* and *L* as a function of *T*.

Figure 8 shows the SANS profiles for a 5 mass % sample (Regime 3) at four different temperatures. This is the fully developed ‘gel’ as visualized in Figure 1. At 70 °C, a linear region (slope ≈ −1) typical for rod-like scatterers is clearly distinguishable. At lower temperatures large-scale aggregates are formed, which dominate the SANS response in the low-*q* region. The value of the exponent *n* increases with decreasing temperature from *n* ≈ 2 (50 °C) to *n* ≈ 3 (25 °C). Such a power-law scattering is indicative of the fractal-like behavior of the system. For scattering from three-dimensional objects the power law exponent magnitude equals (6 − *D*) where *D* is the fractal dimension.

Equation (3) again satisfactorily describes the data over the whole temperature range explored. At low *q*, the slope of the *I*(*q*) curves decreases with increasing temperature, indicating some disassembly of the network. *L* weakly increases and *R* decreases with increasing temperature (see inset). The weak decrease of *L* upon cooling is consistent with some growth of the fibers and a stringer interpenetration that decreases the effective mesh size of the fiber network. The observed reduction of *R* on heating is strikingly similar to what we see in the 0.5 mass % gelator (see Figure 5). We interpret this as meaning that the fibrils are less defective (less branching) in organization at elevated temperatures approaching the ”melting” temperature of the gelator fibers, an effect that arises regardless of the gelator concentration.

## 3. Conclusions

We have studied the thermally reversible gelation of a model peptide by a variety of experimental methods that probe the geometry and relaxation processes of solutions of this gelator molecule over a wide range of spatial and time scales. Our goal was to obtain a holistic view of the gelation process. First, we used simple flow tests to map out a general ‘rheology diagram’ characterizing the dynamic state of the solution, and second, we refined this characterization by NMR measurements, which quantified the fraction of gelator molecules in the self-assembled state. Then rheology measurements were employed to more precisely characterize the progressive change of viscoelasticity of the solution as it was converted to a gel upon increasing the concentration at fixed temperature.

Having characterized the rheological/thermodynamic state of the material, we probed the progressive structural changes by TEM and AFM imaging and small angle neutron scattering and light scattering at higher resolution. We found that the gelator first began to organize into fibers having a molecular size-determined diameter, but was prone to the formation of branching defects that occurred with a greater frequency of occurrence with solution cooling. The fraction of the material in these clusters increased with the gelator concentration. The viscoelasticity and relaxation time of the solution also increased as the gelator concentration increased. These changes were quantified by dynamic light scattering and rheological measurements of the storage and loss moduli of the gelator solutions. Finally, the relaxation time became essentially infinite and a true gel emerged exhibiting nearly frequency independent shear storage and loss moduli. The strength of the gel material was undeniably influenced by the branching defects, as evidenced in the SANS observations, but this effect was not quantified here. Neutron scattering clearly indicates a characteristic scale describing the heterogeneity of the branched fiber network that allows for quantification of the dimensions of structural features observed in TEM and AFM studies, without the problems of material structural distortions that accompany sample preparation in these imaging methods. While we do not expect the pattern of gelation to be universal for all gelators, we do think that our observations do pertain to a wide class of gelators, particularly those not having a proclivity for hierarchical bundling of the primary fibers.

We had originally anticipated that SANS would provide new insight into how the fibers form, but the early stage of fiber growth remains uncertain. Our initial hypothesis was that the fibers would form through bundling of molecular filaments having a diameter on the order of the individual molecules, but we were not able to observe this type of organization by SANS in regime 1, where the gel has not fully formed. Instead, we saw the fibers to be fully organized in this region. We plan to explore this thermodynamic range further in the future to obtain evidence about the initiating structure of fiber formation.

## 4. Experimental

### 4.1. Peptide Synthesis

Molecule **1**, whose chemical topological and geometrical structure is indicated in Scheme 1, was prepared via the standard Fmoc-based solid phase peptide synthesis procedure as described before [46]. Specifically, 1 g of 2-chlorotrityl chloride resin was firstly allowed to swell in anhydrous dichloromethane (DCM). After 20 min bubbling with N_2_, the fully swollen resin was washed with anhydrous dimethylformamide (DMF) (3 × 3 mL). Then the first amino acid Fmoc-Tyr(OPO_3_H_2_) (2 equiv.) was loaded onto resin via the –COOH group by bubbling the resin in a DMF solution containing N,N-diisopropylethylamine (DIPEA) (5 equiv.) for 1 h. After washing with DMF (3 × 3 mL), the resin was blocked by a mixture of DCM/MeOH/DIPEA (80:15:5) for 30 min to deactivate the unreacted sites. Then the resins were subsequently treated with 20% piperidine by mass (in DMF) for 30 min to remove the Fmoc group of Fmoc-Tyr(OPO_3_H_2_), and washed with DMF (5 × 3 mL), coupled with Fmoc-Lys(Boc)–OH (3 equiv.) using DIPEA (5 equiv.) and HBTU (3 equiv.) as the coupling reagent. The Fmoc deprotection (30 min) and amino acid coupling (1 h) steps were then repeated to elongate the desired peptide sequence until Nap was loaded. At the final step, the peptide was cleaved with trifluoroacetic acid (TFA) (10 mL with 1% water by mass) for 2 h and the resulted crude products were precipitated in anhydrous diethyl ether, purified by RP C18 HPLC (Waters, Parsippany, NJ, USA) and freeze dried to produce the white powder product of **1**.

### 4.2. Hydrogel Preparation

One hundred milligrams of molecule **1** was first mixed at room temperature with 1.75 mL ultra-pure water and 150 μL of 1 mol/L NaOH resulting in a suspension. The suspension turned into a clear viscous solution after incubating in a water bath at 85 °C. When the solution was allowed to cool down to room temperature, it resulted in a 5 mass % hydrogel. Gels were also prepared at other concentrations (0.5, 1, and 2) mass % with the appropriate amounts of peptide and solvent.

### 4.3. Measurement Methods

#### 4.3.1. Proton NMR

^1^H-NMR was conducted on a GSX-270 Jeol 270 MHz NMR spectrometer (JEOL ,Tokyo, Japan) [47]. Samples were prepared by dissolving **1** in D_2_O at various concentrations with additional NaOH to adjust the pH to 7.4, loaded into 5 mm NMR tubes and sealed. The measurements were conducted at certain temperatures ranging from 25 °C to 70 °C with 10 min of equilibration at each desired temperature. Other experimental parameters: 10 μs excitation pulse, 15 Hz spinning, and 8 to 16 scans.

#### 4.3.2. Dynamic Light Scattering (DLS)

Dynamic light scattering measurements were performed on a PDExpert Multi-Angle Light Scattering platform (Precision Detectors, Inc., Bellingham, MA, USA). A HeNe laser (wavelength: 685 nm, power: 30 mW was employed. Control of the system as well as data processing and storage were made by a PrecisionDeconvolve32 dynamic light scattering instrument. The sample temperature was controlled (from 0 °C to 80 °C) by a Peltier controller. DLS measurements were made on each peptide solution in the temperature range from 4 °C to 70 °C.

#### 4.3.3. Small Angle Neutron Scattering (SANS)

SANS measurements were performed on the 30 m instrument (NG-3,) [48] at the Center for Neutron Research (NCNR), National Institute of Standards and Technology (NIST), Gaithersburg, MD, USA. Samples were prepared by dissolving molecule **1** in D_2_O and using 1 mol/L NaOH (prepared with D_2_O) to adjust the pH to 7.4. Solutions were transferred to titanium sample cells with 30 mm diameter quartz windows and 2 mm path length. All samples were allowed to pre-equilibrate overnight at room temperature prior to the scattering measurement. A monochromated neutron beam (wavelength λ = 0.6 nm (6 Å) at 1 m and 4 m sample to detector distances, and λ = 0.84 nm (8.4 Å) at 13 m sample to detector distance with focusing lenses) was used with a wavelength spread (Δλ/λ) of 0.14. The scattered neutrons were captured on a 64 cm × 64 cm 2D detector. The scattering wave vector *q* (*q* = (4π/λ) sin (θ/2), where θ is the scattering angle) was varied in the range 0.04 nm^−1^ < *q* < 4 nm^−1^. Raw data were corrected for background electronic noise and scattering noise radiation, detector inhomogeneity and sensitivity, and empty cell scattering. Intensities were normalized to an absolute scale relative to the main beam transmission measurements through the sample and were reduced according to published protocol [49]. Incoherent background correction was made using a standard subtraction procedure [50]. The error bars of the data points for all SANS plots are comparable to the size of the symbols.

#### 4.3.4. Transmission Electron Microscopy (TEM)

A drop of peptide solution (approximate 5 μL) of known concentration was transferred to a carbon film coated copper grid (200 square mesh), blotted with filter paper, and negative stained with uranium acetate (2% by mass). All samples were prepared at room temperature. Imaging was carried out in a Tecnai TF30 transmission electron microscope (TEM) (FEI, Hillsboro, OR, USA) equipped with a Gatan Ultrascan 1000 CCD camera (Gatan, Pleasaton, CA, USA).

#### 4.3.5. Atomic Force Microscopy (AFM)

A commercial AFM instrument (Multimode/PicoForce system with NS-V controller, Bruker Nano-surface, Inc., Santa Barbara, CA, USA) was used to image air-dried samples on freshly cleaved mica surface. A drop of peptide solution (≈5 μL) was allowed to stay on a mica surface for 5 min before being washed with 3 × 1 mL of Milli-Q water and dried in a nitrogen stream. Silicon cantilevers by Bruker (FESP, ≈75 kHz, 2.8 N·m^−1^) were used to acquire images of the supramolecular assemblies.

#### 4.3.6. Oscillatory Rheological Measurements

Rheological measurements were carried out on an ARES-G2 rheometer (TA instruments, New Castle, DE, USA) using parallel plate geometry and a 25 mm stainless steel tool. The rheometer was pre-equilibrated at 4 °C. Peptide solutions or hydrogels were also pre-equilibrated at 4 °C, and a 300 μL aliquot was transferred to the rheometer plate. A gap of 500 μm was used for all measurements. Standard mineral oil (from Sigma-Aldrich M3516-1L) was placed around the sample to prevent water evaporation over time. In a typical experiment, the temperature was maintained (e.g., 20 °C) for 1 h during a dynamic time sweep experiment performed at an angular frequency of 6 rad·s^−1^ and a strain of 0.2%. Frequency sweeps from 0.1 rad·s^−1^ to 100 rad·s^−1^ at a strain of 0.2% and amplitude sweeps from 0.01% to 100% strain at a frequency of 6 rad·s^−1^ were subsequently performed to assess the frequency dependence and the linear viscoelastic region, respectively.
